# Body fat changes and risk of new onset of hypertension and hyperlipidaemia among Korean adults: A longitudinal study

**DOI:** 10.1016/j.clinme.2025.100293

**Published:** 2025-02-11

**Authors:** Jinyoung Shin, Sang-Hyun Park, Jae Hoon Cho, Tae-Eun Kim

**Affiliations:** a Department of Family Medicine, Konkuk University Medical Center, Konkuk University School of Medicine, Seoul 05030, South Korea; b Department of Clinical Pharmacology, Konkuk University Medical Center, Seoul 05030, South Korea; c Department of Otorhinolaryngology-Head and Neck Surgery, Konkuk University Medical Center, Konkuk University School of Medicine, Seoul 05030, South Korea

**Keywords:** Body fat, Hypertension, Hyperlipidaemia, Regular health screening

## Abstract

**Background:**

This study examined the association between changes in body fat, body mass index (BMI), and the risk of newly developed hypertension and hyperlipidaemia in the general population.

**Methods:**

This longitudinal study included 17,598 individuals without prior hypertension and hyperlipidaemia who underwent at least three health examinations between January 2015 and December 2022. Body fat was classified as decreased (≥ 5%), stable (within 5%), and increased (≥ 5%) using bioelectrical impedance analysis. BMIs were categorised as healthy weight/underweight (BMI < 23), overweight (23 ≤ BMI < 25), and obesity (BMI ≥ 25). Hypertension and hyperlipidaemia were identified through self-reported medication use or clinical measurements.

**Results:**

Increases in BMI or body fat were associated with higher incidence rates of hypertension and hyperlipidaemia. Decreased body fat was associated with a lower risk of hypertension in the overweight (odds ratio: 0.638, 95% confidence interval: 0.464–0.876) and obese groups (0.724, 0.577–0.909). Individuals with healthy weight/underweight with increased body fat had a higher incidence of hyperlipidaemia than individuals with overweight with decreased body fat (87.2 vs 66.4 per 1,000 people). Compared to the stable body fat group, increased body fat raised the risk of hyperlipidaemia (healthy weight/underweight: 1.522, 1.248–1.855; overweight: 1.278, 1.032–1.583; and obesity: 1.214, 1.028–1.433). Individuals living with overweight with decreased body fat demonstrated a lower risk of hyperlipidaemia (0.546, 0.400–0.747).

**Conclusions:**

Increased body fat was associated with a higher risk of hyperlipidaemia, even within the same BMI category. Decreasing body fat, particularly in individuals living with overweight, is associated with a lower risk of hypertension and hyperlipidaemia.

## Highlights

• Individuals with overweight and obesity with reduced body fat have a lower risk of hypertension than those with stable body fat.
• Increased body fat elevates hyperlipidaemia risk across all BMI categories compared to stable body fat.
• Individuals with overweight with reduced body fat have a lower hyperlipidaemia risk than those with stable fat.

## Graphical abstract

**Figure:**
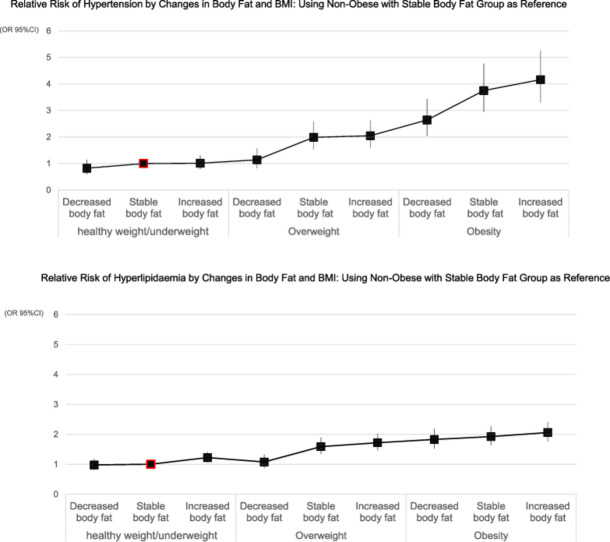

## Introduction

Hypertension is a pervasive global comorbidity and one of the most significant factors for cardiovascular disease, chronic kidney disease and stroke.^1^ Hypertension was estimated to affect 33% of adults aged 30–79 worldwide in 2019.^2^ In 2022, hypertension affected 29.7% of the Korean population, with 33.1% of males and 26.5% of females experiencing this condition.^3^ This represents a significant increase from 2007 when the overall prevalence was 20.3% (21.4% for males and 19.2% for females). The surge in hypertension cases has been attributed to various modifiable risk factors, notably obesity and hyperlipidaemia. These factors are crucial for the progression of atherosclerosis and related cardiovascular disorders.^3^^,^^4^

Obesity is defined by body mass index (BMI), calculated as weight in kilograms divided by the square of height in meteres, and is a key measure for identifying individuals at risk of metabolic health complications, including elevated blood pressure and altered lipid profiles.^5^ Obesity rates, defined as BMI ≥ 25, have been steadily increasing, with data showing rates between 30.9% in 2014 and 37.2% in 2022 in Korea.^3^^,^^6^ This trend suggests that the increasing incidence of obesity may contribute to the increased prevalence of hypertension and hyperlipidaemia.^7^

The aetiology of obesity is multifaceted, with biological, environmental and lifestyle factors promoting high-calorie diets and sedentary behaviours.^4^ Although BMI is widely used to classify obesity, the assessment of obesity using only BMI has several limitations, particularly the inability to differentiate between fat and lean mass.^8^ Moreover, BMI does not account for variations in fat distribution, which can vary significantly according to age, sex and ethnicity.^9^, ^10^, ^11^ For instance, Asians tend to have a higher percentage of body fat at a given BMI than Caucasians, leading to differences in the cardiometabolic risk profiles of these populations.^12^

The relationship between obesity and hypertension has been well established, with estimates suggesting that obesity is responsible for 65–78% of essential hypertension cases.^5^ Hyperlipidaemia, particularly elevated levels of low-density lipoprotein (LDL) cholesterol, is closely associated with excessive body fat and plays a central role in the development of atherosclerosis. Previous research has established BMI as a critical predictor of hypertension, whereas body fat content has been identified as a significant predictor of hyperlipidaemia.^13^ However, despite the clear associations between obesity, hypertension and hyperlipidaemia, research is limited because the obesity status, including BMI and body fat content, can change over time. This gap underscores the need for a focused analysis of how changes in body fat over time might influence the incidence of these conditions based on BMI.

Accordingly, this study aimed to examine the effects of changes in body fat measured at different times on the onset of hypertension and hyperlipidaemia, according to BMI categories. In this study, we sought to determine whether efforts to reduce body fat could mitigate the risk of developing hypertension and hyperlipidaemia.

## Materials and methods

### Study design and participants

We recruited 22,631 individuals aged 20 years or older who underwent regular health examinations at least three times with complete data at a tertiary hospital health examination centre in Seoul between January 2015 and December 2022. Individuals with a prior diagnosis of hypertension taking antihypertensive medication (n = 1,639), hyperlipidaemia taking lipid-lowering medication (n = 2,691), and both (n = 703) were excluded at baseline. The final study population (n = 17,598) was enrolled.

The Institutional Review Board of the Clinical Research Ethics Committee of the tertiary hospital granted approval for study exemption, as all data were collected without identifiable information (KUMC 2022-12-010). The requirement for informed consent was waived.

### Definition of newly developed hypertension and hyperlipidaemia

Newly developed hypertension was defined as either a physician-reported prescription for antihypertensive medication after a previous health screening, measured systolic blood pressure of 140 mmHg or higher, or diastolic blood pressure of 90 mmHg or higher. Blood pressure was measured once by a trained nurse while the subject was in a seated position. Similarly, newly developed hyperlipidaemia was defined as a physician-reported prescription for lipid-lowering agents or a total cholesterol level ≥ 240 mg/dL. Lipid profiles were measured after a minimum 12 h overnight fast using a Hitachi-7600 blood analyser (Hitachi, Tokyo, Japan).

### Body fat changes

Body fat was measured using bioelectrical impedance analysis (BIA; InBody 770© (Biospace, Seoul, South Korea)), a validated method for assessing body composition.^14^ The measurement was conducted while the participants maintained an upright position with their feet separated and elbows extended to prevent body contact for approximately 1 min. The bare feet made contact with the base electrodes at the heels and forefeet while the participants grasped the handle electrodes, making direct contact with additional electrodes on the thumbs and forefingers.^15^^,^^16^ Participants wore a light attire without footwear. Differences in total body fat and visceral fat were calculated by subtracting the values from the first and second examinations. Participants were divided into three groups based on the change in total body fat between the first and second health examinations: those with a decrease in total body fat of more than 5% were designated as the ‘decreased body fat’ group, those with changes within 5% were classified as the ‘stable body fat’ group, and those with an increase of more than 5% were categorised as the ‘increased body fat’ group.

### Other study measurements

Waist circumference (cm) was measured at the midpoint between the lower border of the rib cage and the upper border of the iliac crest. A self-administered questionnaire was used to collect data on each participant’s medical history (hypertension, hyperlipidaemia, ischaemic heart disease, myocardial infarction, stroke and cancer), medication use, smoking and alcohol consumption habits, physical activity and marital status. Smoking status was defined as current smoking status. Alcohol consumption was defined as regular drinking (more than once per week). Regular exercise was defined as engaging in moderate-intensity physical activity more than once a week. Marital status was categorised as married/with a partner or divorced/widowed/unmarried.

### Statistical analysis

This analysis aimed to determine an association between changes in body fat and the incidence of hypertension and hyperlipidaemia based on BMI categories. The initial BMI categories were stratified into three groups: healthy weight/underweight (BMI < 23), overweight (23 ≤ BMI < 25), and obesity (BMI ≥ 25) according to the WHO Asia-Pacific classification and the diagnosis of obesity on the Korean Society for the Study of Obesity.^6^^,^^17^ Continuous variables were presented as the mean ± standard deviation, while categorical variables were presented as the number of subjects (percentage). Changes in weight, BMI, waist circumference, body fat and visceral fat were calculated by subtracting the values from the second visit from those from the first visit. Statistical differences were assessed using two methods. First, the characteristics of participants were compared among the three categories according to body fat change: decreased, stable and increased body fat. Second, these were compared across the BMI categories: healthy weight/underweight, overweight and obesity, using ANOVA and chi-square tests. The incidence rates of hypertension and hyperlipidaemia per 1,000 individuals were calculated. Odds ratios (ORs) with 95% confidence intervals (CIs) for the risk of newly diagnosed hypertension and hyperlipidaemia were estimated using univariate and multivariate logistic regression analyses adjusted for age, sex, initial BMI, comorbidities, smoking status, alcohol consumption and regular exercise. The reference group consisted of participants with stable body fat in the same BMI category. All statistical analyses were conducted using the R statistical software (version 4.3.1; R Foundation for Statistical Computing, Vienna, Austria).

## Results

Baseline characteristics, including lifestyle habits and comorbidities, are presented according to the three BMI categories and body fat changes (Table 1). Among the total 17,598 participants (9,907 males (56.3%) and 7,691 females (43.7%)), statistically significant differences in weight, BMI, waist circumference, total body fat and visceral fat, and their changes were observed according to changes in body fat within the three groups classified by BMI criteria. Groups with healthy weight/underweight, overweight, and obesity revealed statistically significant differences in all variables except for changes in waist circumference. Additionally, significant differences in smoking status, alcohol consumption, physical activity levels, and history of cardiovascular disease and cancer were observed across the BMI categories.

**Table 1:** Baseline characteristics of study population (n = 17,598).

	Healthy weight/underweight (BMI < 23) (n = 8,867)				Overweight (23 ≤ BMI < 25) (n = 4,124)				Obesity (25 ≤ BMI) (n = 4,607)				*P*-value^1^
	Decreased body fat	Stable body fat	Increased body fat	*P*-value	Decreased body fat	Stable body fat	Increased body fat	*P*-value	Decreased body fat	Stable body fat	Increased body fat	*P*-value	
N (%)	1,793 (20.2)	2,443 (27.6)	4,631 (52.2)		934 (22.6)	1,321 (32.0)	1,869 (45.3)		1,053 (22.9)	1,563 (33.9)	1,991 (43.2)		
Age (year)	41.9 ± 9.6	43.2 ± 9.1	42.4 ± 9.4	0.002	44.8 ± 9.4	46.5 ± 9.7	45.8 ± 10.2	0.057	44.7 ± 9.1	45.6 ± 8.9	46.15 ± 10.2	0.124	<0.001
Weight (kg)	55.6 ± 7.7	56.5 ± 7.2	58.2 ± 8.1	<0.001	67.3 ± 7.6	68.1 ± 7.4	70.6 ± 7.7	<0.001	75.6 ± 9.9	78.4 ± 9.7	81.0 ± 10.7	<0.001	<0.001
Δ Weight (kg)	−1.5 ± 1.9	0.01 ± 1.3	1.8 ± 2.1	<0.001	−2.1 ± 2.5	-0.07 ± 1.6	1.9 ± 2.3	<0.001	−3.3 ± 3.4	-0.23 ± 1.9	1.9 ± 2.9	<0.001	<0.001
BMI (kg/m^2^)	20.1 ± 1.6	20.7 ± 1.5	21.1 ± 1.8	<0.001	23.1 ± 1.1	23.8 ± 0.8	24.5 ± 1.0	<0.001	25.9 ± 2.1	27.0 ± 2.1	27.9 ± 2.5	<0.001	<0.001
Δ BMI (kg/m^2^)	−0.7 ± 0.7	−0.06 ± 0.5	0.6 ± 0.8	<0.001	−0.8 ± 0.9	−0.1 ± 0.6	0.6 ± 0.8	<0.001	−1.3 ± 1.2	−0.2 ± 0.7	0.6 ± 1.0	<0.001	<0.001
WC (cm)	71.7 ± 6.3	73.4 ± 6.3	74.4 ± 6.7	<0.001	81.1 ± 5.5	83.0 ± 5.1	84.8 ± 5.3	<0.001	87.8 ± 7.0	90.9 ± 6.5	92.8 ± 7.2	<0.001	<0.001
Δ WC (cm)	−2.3 ± 3.8	−0.8 ± 3.5	0.7 ± 3.8	<0.001	−2.6 ± 3.9	−0.6 ± 3.2	1.2 ± 3.6	<0.001	−3.3 ± 4.3	−0.7 ± 3.3	1.1 ± 3.7	<0.001	0.655
Fat (g)	11.8 ± 3.0	13.9 ± 2.9	15.1 ± 3.7	<0.001	15.0 ± 2.9	17.5 ± 2.6	19.1 ± 3.4	<0.001	19.5 ± 4.3	22.3 ± 4.7	24.7 ± 5.8	<0.001	<0.001
Δ Fat (g)	-1.8 ± 1.1	0.04 ± 0.4	2.4 ± 1.8	<0.001	-2.3 ± 1.5	0.05 ± 0.5	2.7 ± 1.8	<0.001	-3.0 ± 2.1	0.05 ± 0.63	3.3 ± 2.2	<0.001	<0.001
Visceral fat (g)	5.1 ± 2.4	6.4 ± 2.5	6.9 ± 2.5	<0.001	7.5 ± 2.7	9.4 ± 2.5	9.7 ± 2.5	<0.001	9.5 ± 2.8	11.4 ± 2.7	12.1 ± 2.9	<0.001	<0.001
Δ Visceral fat (g)	−1.3 ± 1.8	−0.2 ± 1.4	0.9 ± 2.5	<0.001	−2.2 ± 2.3	−0.7 ± 1.9	0.02 ± 3.5	<0.001	−2.4 ± 2.2	−0.6 ± 1.8	0.4 ± 2.3	<0.001	<0.001
Smoking, N (%)	227 (12.7)	228 (9.3)	531 (11.5)	<0.001	199 (21.3)	282 (21.3)	394 (21.1)	0.491	257 (24.4)	417 (26.7)	535 (26.9)	0.542	<0.001
Alcohol drinking, N (%)	1,188 (66.3)	1,564 (64)	3,083 (66.6)	0.089	745 (79.8)	997 (75.5)	1,428 (76.4)	0.048	838 (79.6)	1,259 (80.6)	1,553 (78.0)	0.169	<0.001
Exercise, N (%)	1,097 (61.2)	1,597 (65.4)	3,103 (67.0)	<0.001	506 (54.2)	778 (58.9)	1,131 (60.5)	0.006	569 (54.0)	965 (61.7)	1,222 (61.4)	<0.001	<0.001
CVD, N (%)	4 (0.2)	6 (0.2)	10 (0.2)	0.969	3 (0.3)	12 (0.9)	22 (1.2)	0.077	13 (1.2)	11 (0.7)	19 (1.0)	0.380	<0.001
Malignancy, N (%)	56 (3.1)	72 (2.9)	152 (3.3)	0.742	20 (2.1)	26 (2.0)	62 (3.3)	0.037	23 (2.2)	32 (2.0)	61 (3.1)	0.116	0.041
Marital status, N (%)				<0.001				0.030				0.043	<0.001
Married/with partner	1,413 (78.8)	2,084 (85.3)	3,830 (82.7)		805 (86.2)	1,181 (89.4)	1,617 (86.5)		931 (88.4)	1,419 (90.8)	1,758 (88.3)		
Divorced/widowed /unmarried	380 (21.2)	359 (14.7)	801 (17.3)		129 (13.8)	140 (10.6)	252 (13.5)		122 (11.6)	144 (9.2)	233 (11.7)		

Data are presented as N (%) for categorical variables or mean ± standard deviation (SD) for continuous variables. BMI: baseline value of body mass index, WC: waist circumference. *P*-values^1^ were evaluated a significant difference across the BMI category.

Cardiovascular diseases (CVD) were classified as ischaemic heart disease, stroke and myocadial infarction.

Table 2 presents the clinical variables associated with hypertension and hyperlipidaemia according to the body fat changes and BMI categories. As BMI increased, the mean values of systolic blood pressure and total cholesterol, triglyceride and LDL cholesterol levels increased, while that of high-density lipoprotein (HDL) cholesterol decreased. Changes in body fat were strongly correlated with changes in triglyceride levels across all BMI categories. In the group of healthy weight/underweight, changes in diastolic blood pressure and HDL cholesterol levels were associated with changes in body fat.

**Table 2:** Clinical variables of study population.

	Healthy weight/underweight (BMI <23)				Overweight (23 ≤ BMI < 25)				Obese (25 ≤ BMI)				*P*-value^1^
	Decreased body fat	Stable body fat	Increased body fat	*P*-value	Decreased body fat	Stable body fat	Increased body fat	*P*-value	Decreased body fat	Stable body fat	Increased body fat	*P*-value	
SBP	128.2 ± 12.7	128.7 ± 13.1	130.5 ± 15.4	0.273	128.4 ± 15.2	129.4 ± 13.6	130.2 ± 14.1	0.552	130.5 ± 13.0	131.4 ± 13.2	133.1 ± 12.6	0.074	<0.001
Δ SBP	4.5 ± 11.4	4.1 ± 13.7	7.1 ± 13.7	0.066	2.82 ± 13.9	4.4 ± 12.8	6.0 ± 13.1	0.210	4.2 ± 12.3	6.0 ± 12.0	7.4 ± 12.8	0.098	0.165
DBP	78.5 ± 9.7	80.5 ± 11.0	81.6 ± 12.1	0.344	79.3 ± 11.9	81.1 ± 11.0	80.4 ± 11.5	0.612	80.8 ± 11.4	81.0 ± 11.0	81.9 ± 10.6	0.257	0.189
Δ DBP	1.4 ± 9.1	2.2 ± 10.3	5.5 ± 11.7	0.011	2.7 ± 10.3	4.2 ± 10.3	4.8 ± 10.3	0.499	4.0 ± 11.3	4.7 ± 9.6	5.6 ± 10.4	0.236	0.036
Heart rate	74.4 ± 10.3	74.4 ± 10.9	75.8 ± 12.4	0.298	77.9 ± 12.5	76.0 ± 13.6	73.7 ± 12.8	0.077	71.8 ± 11.1	74.3 ± 12.4	75.1 ± 10.7	0.259	0.196
Δ Heart rate	0.3 ± 10.4	0.4 ± 9.3	0.7 ± 10.2	0.758	2.3 ± 8.9	2.4 ± 10.3	0.3 ± 11.0	0.052	-0.8 ± 10.4	2.2 ± 9.7	1.7 ± 9.3	0.707	0.287
Total-C	208.3 ± 52.8	219.2 ± 46.1	220.9 ± 49.8	0.518	218.3 ± 48.7	228.8 ± 40.9	232.6 ± 43.0	0.279	230.7 ± 43.6	241.2 ± 34.8	238.7 ± 35.8	0.820	<0.001
Δ Total-C	7.9 ± 44.0	18.3 ± 34.5	19.8 ± 38.2	0.443	17.8 ± 32.9	24.6 ± 33.3	27.4 ± 34.6	0.313	20.0 ± 36.9	28.4 ± 32.3	29.4 ± 31.6	0.375	<0.001
Triglyceride	94.8 ± 52.9	109.4 ± 61.6	107.6 ± 58.2	0.876	129.6 ± 65.2	141.2 ± 103.5	158.3 ± 115.3	0.080	154.0 ± 94.2	175.7 ± 141.1	191.9 ± 179.0	0.145	<0.001
Δ Triglyceride	−10.1 ± 52.5	1.7 ± 59.9	9.6 ± 49.2	0.027	−13.6 ± 60.3	0.9 ± 86.2	26.1 ± 83.7	0.001	−13.2 ± 76.3	1.6 ± 97.0	27.6 ± 146.8	0.016	<0.001
HDL- C	73.0 ± 17.0	69.8 ± 17.2	70.4 ± 18.8	0.944	59.2 ± 13.7	60.6 ± 14.4	59.0 ± 14.3	0.255	55.6 ± 15.6	54.6 ± 14.1	52.9 ± 11.1	0.082	<0.001
Δ HDL-C	7.9 ± 9.7	6.4 ± 8.8	4.0 ± 10.6	0.002	5.5 ± 7.3	3.9 ± 8.0	3.1 ± 8.4	0.214	4.2 ± 9.0	2.8 ± 7.3	2.0 ± 7.6	0.150	<0.001
LDL-C	127.9 ± 46.2	137.4 ± 41.7	139.3 ± 44.6	0.451	136.8 ± 42.0	144.1 ± 38.2	148.4 ± 39.7	0.207	139.9 ± 37.4	151.6 ± 32.7	148.8 ± 33.1	0.777	<0.001
Δ LDL-C	4.9 ± 38.9	13.9 ± 30.5	15.1 ± 33.5	0.480	13.4 ± 29.3	20.0 ± 28.7	21.9 ± 32.1	0.423	13.3 ± 30.5	21.9 ± 28.9	24.0 ± 28.6	0.157	<0.001

SBP, systolic blood pressure; DBP, diastolic blood pressure; Total-C, total cholesterol; HDL-C, HDL-cholesterol; and LDL-C, LDL- cholesterol.

Δ was calculated by subtracting the values from the first and second examinations.

*P*-value^1^’s were evaluated a significant difference across the BMI category.

The median follow-up period was 2.26 years (interquartile range: 1.98–3.80). During the observation period, 1,624 patients (9.2% of the total subjects) developed hypertension (Table 3). The incidence rate of hypertension in the group with healthy weight/underweight was 3.7%, which increased to 10.5% in the group living with overweight and 18.7% in the group living with obesity. Changes in body fat did not significantly affect the risk of hypertension in the non-obese group. However, in the overweight and obesity groups, individuals with decreased body fat showed a lower risk of hypertension than those with stable body fat within the same BMI category. Incidence rates (IRs per 1,000 persons) of hypertension increased in conjunction with higher BMI and increased body fat.

**Table 3:** Odd ratios of newly developed hypertension according to the body fat changes and BMI category.

	N	Event	IR (1,000 person)	Crude	Age and sex adjusted	Age, sex, initial BMI, and comorbidity	Age, sex, initial BMI, comorbidity and lifestyle
Healthy weight/underweight							
Decreased body fat	1,793	57	31.79	0.868 (0.619–1.218)	0.865 (0.610–1.227)	0.857 (0.603–1.216)	0.863 (0.608–1.226)
Stable body fat	2,443	89	36.43	1 (ref.)	1 (ref.)	1 (ref.)	1 (ref.)
Increased body fat	4,631	178	38.44	1.057 (0.816–1.370)	1.042 (0.796–1.363)	1.007 (0.769–1.319)	1.012 (0.772–1.325)
Overweight							
Decreased body fat	934	65	69.59	0.588 (0.434–0.798)	0.612 (0.447–0.838)	0.640 (0.466–0.880)	0.638 (0.464–0.876)
Stable body fat	1,321	149	112.79	1 (ref.)	1 (ref.)	1 (ref.)	1 (ref.)
Increased body fat	1,869	217	116.11	1.033 (0.828–1.289)	1.034 (0.821–1.303)	1.011 (0.801–1.277)	1.013 (0.802–1.280)
Obesity							
Decreased body fat	1,053	150	142.45	0.714 (0.576–0.885)	0.728 (0.583–0.909)	0.731 (0.583–0.918)	0.724 (0.577–0.909)
Stable body fat	1,563	295	188.74	1 (ref.)	1 (ref.)	1 (ref.)	1 (ref.)
Increased body fat	1,991	424	212.96	1.163 (0.985–1.373)	1.103 (0.928–1.312)	1.090 (0.913–1.303)	1.097 (0.918–1.311)

IR, incidence rate, BMI, body mass index.

Among the 1,921 patients (10.9%) newly diagnosed with hyperlipidaemia (Table 4), the incidence rates were 7.6%, 12.1%, and 16.3% in individuals living with healthy weight, overweight and obesity, respectively. Regardless of the BMI classification, participants with increased body fat exhibited a higher risk of hyperlipidaemia than those with stable body fat (within a 5% change). Notably, the effect of increased body fat on the development of hyperlipidaemia was prominent in the group with healthy weight/underweight (ORs: 1.522, 95% CI: 1.248–1.855). In the group living with overweight, individuals with decreased body fat had a lower risk of hyperlipidaemia than those with stable body fat levels. Additionally, the incidence per 1,000 individuals in the group living with overweight with decreased body fat (IR: 66.38) was significantly lower than that in the group of healthy weight/underweight with increased body fat (IR: 87.24).

**Table 4:** Odd ratios of newly developed hyperlipidaemia according to the body fat changes and BMI category.

	N	Event	IR (1,000 people)	Crude	Age and sex adjusted	Age, sex, initial BMI, and comorbidity	Age, sex, initial BMI, comorbidity and lifestyle
Healthy weight/underweight							
Decreased body fat	1,793	116	64.70	1.043 (0.812–1.338)	1.115 (0.864–1.438)	1.114 (0.862–1.438)	1.116 (0.864–1.442)
Stable body fat	2,443	152	62.22	1 (ref.)	1 (ref.)	1 (ref.)	1 (ref.)
Increased body fat	4,631	404	87.24	1.441 (1.187–1.748)	1.518 (1.246–1.850)	1.521 (1.248–1.855)	1.522 (1.248–1.855)
Overweight							
Decreased body fat	934	62	66.38	0.512 (0.377–0.696)	0.536 (0.393–0.730)	0.542 (0.397–0.741)	0.546 (0.400–0.747)
Stable body fat	1,321	161	121.88	1 (ref.)	1 (ref.)	1 (ref.)	1 (ref.)
Increased body fat	1,869	275	147.14	1.243 (1.009–1.531)	1.266 (1.025–1.564)	1.270 (1.025–1.572)	1.278 (1.032–1.583)
Obesity							
Decreased body fat	1,053	142	134.85	0.887 (0.726–1.082)	0.900 (0.735–1.102)	0.912 (0.743–1.120)	0.922 (0.750–1.133)
Stable body fat	1,563	242	154.83	1 (ref.)	1 (ref.)	1 (ref.)	1 (ref.)
Increased body fat	1,991	367	184.33	1.247 (1.062–1.464)	1.213 (1.030–1.428)	1.212 (1.026–1.430)	1.214 (1.028–1.433)

IR, incidence rate; BMI, body mass index.

## Discussion

This study investigated the association between changes in body fat according to BMI categories, and the risk of newly developed hypertension and hyperlipidaemia in the general population. We found an overall positive correlation between an increased BMI and a higher risk of hypertension or hyperlipidaemia. Notably, individuals in the overweight and obese groups who experienced a decrease in body fat had a lower risk of hypertension than those whose body fat levels remained stable in the same BMI category. Conversely, increased body fat was associated with a higher risk of hyperlipidaemia across all BMI categories compared to stable body fat levels. Furthermore, even in the group living with overweight, individuals who experienced a decrease in body fat had a lower risk of developing hyperlipidaemia than those who maintained their body fat. Even in the group of healthy weight/underweight, individuals with increased body fat showed a higher incidence of hyperlipidaemia and a greater risk of developing hyperlipidaemia than those in the overweight group with decreased body fat. These findings underscore the importance of body fat changes in mitigating the risks of hypertension or hyperlipidaemia.

Obesity-associated effects in lipoprotein metabolism, characterised by increased triglycerides, decreased HDL cholesterol, and either normal or slightly elevated LDL cholesterol, can be partly attributed to decreased mRNA expression levels of lipoprotein lipase in adipose tissues, impairing the lipolysis of TG-rich lipoproteins.^5^ Although we did not measure it, the changes in triglyceride and HDL cholesterol levels could result in the development of hypertension or hyperlipidaemia because of the significant differences in these variables in this study. Additionally, obesity is associated with a pro-inflammatory state through elevated inflammatory mediators and reduced adiponectin levels, contributing to vascular and endothelial dysfunction and initiating hypertension and atherosclerotic events.^6^ Although we did not measure any inflammation markers due to the limitations of using data obtained from routine health examinations, it is presumed that increased inflammation may have contributed, as unhealthy lifestyles and the prevalence of cardiovascular disease are associated with higher BMI and body fat levels.^18^^,^^19^

A significant correlation was noted between changes in body fat and hyperlipidaemia, defined by total cholesterol levels, reinforcing the relationship between body fat alterations and hyperlipidaemia, as evidenced in both this study and a large-scale study involving over 3.6 million participants from the Korean National Health Insurance System cohort.^20^ A study involving 253 participants without metabolic syndrome who underwent repeat CT showed that changes in visceral fat were associated with an increased risk of metabolic syndrome irrespective of changes in overall weight or initial demographic factors.^21^ While our study did not separate the effect of general or visceral when assessing the risk of hypertension and hyperlipidaemia, further research should consider specifically exploring visceral fat.

Weight has been known for a useful indicator of the risk of diabetes mellitus development in large populations, as demonstrated in a study of 4,818 Korean adults over 5 years.^22^ However, few studies have examined the association between weight and hypertension or hyperlipidaemia. Employing body fat measurements could provide more sensitive predictions and interventions to manage the risks associated with hypertension and hyperlipidaemia, like as this study.

It is essential to consider the adiposity levels according to BMI categories on the cardiovascular risk. In a study of 6,997 Chinese patients with type 2 diabetes mellitus, visceral obesity (visceral fat area ≥ 100 cm^2^) measured using BIA in conjunction with BMI was associated with an increased 10-year risk of atherosclerotic cardiovascular disease.^23^ This finding underscores the importance of coupling BMI and adiposity measures in obesity assessment. Notably, individuals with normal BMI but high visceral obesity exhibited a higher prevalence of hypertension and increased total cholesterol levels, similar to the results observed in our study. This aligns with other studies that utilised BIA for measurement, reinforcing the relevance of comprehensive obesity assessment tools.

This study has several limitations. First, the thresholds for significant changes in fat mass indices were arbitrarily set at 5% owing to the absence of standardised cut-off values, which restricts our ability to delineate precise associations, although BMI and body fat distribution may fluctuate over time. Additionally, the intervals between measurements varied among participants, necessitating consideration of the differences in measurement periods, rather than assuming linear changes. Second, our analysis did not differentiate between intentional and unintentional changes in obesity status through healthy lifestyle adjustments with standardised physical activity or starvation, potentially confounding the impact of these factors on the studied conditions. Third, we need to account for a broader range of known risk factors for hypertension and hyperlipidaemia, as this study used health examination data. Finally, this study was conducted in the Korean population. Therefore, careful consideration is required before these findings can be applied to other countries or ethnic groups with different body compositions. Additionally, the participants had undergone repeated health screenings, indicating that they were relatively healthy and had a higher interest in their health. Therefore, caution is needed when generalising these results to broader populations.

In conclusion, changes in body fat were found to influence the risk of newly diagnosed hypertension and hyperlipidaemia. This study supports that a reduction in body fat, particularly among overweight individuals, was associated with a decreased risk of both conditions. It is emphasising the importance of considering body fat levels as well as BMI or body weight, as part of a comprehensive approach to mitigating cardiometabolic risk. Further research is necessary to include more specific markers of obesity, such as waist:hip ratio and visceral fat area, and to clarify these associations and refine the strategies for cardiovascular risk reduction, particularly in diverse populations.

## Funding

This research was funded by Konkuk University Medical Centre. The funders had no role in the design of the study, collection of data, analyses, interpretation of data, writing of the manuscript, or the decision to publish the results.

## Data availability statement

The data that support the findings of this study are available on request from the corresponding author.

## Ethics approval and consent to participate

The study was conducted in accordance with the guidelines of the Declaration of Helsinki and approved by the Institutional Review Board of the University Medical Center (KUMC 2022-12-010. The requirement for informed consent was waived.

## Author contributions

**JS:** conceptualization, methodology, formal analysis, and writing—original draft. **SHP:** conceptualization, methodology, data curation and formal analysis. **JHC:** conceptualization, methodology, and writing—review and editing. **TEK:** conceptualization, methodology, formal analysis, writing—review and editing, and funding acquisition.

All authors have read and agreed to the published version of the manuscript.

## Declaration of competing interest

The authors declare that they have no known competing financial interests or personal relationships that could have appeared to influence the work reported in this paper.
